# Additive manufacture of complex 3D Au-containing nanocomposites by simultaneous two-photon polymerisation and photoreduction

**DOI:** 10.1038/s41598-017-17391-1

**Published:** 2017-12-07

**Authors:** Qin Hu, Xue-Zhong Sun, Christopher D. J. Parmenter, Michael W. Fay, Emily F. Smith, Graham A. Rance, Yinfeng He, Fan Zhang, Yaan Liu, Derek Irvine, Christopher Tuck, Richard Hague, Ricky Wildman

**Affiliations:** 1 Centre for Additive Manufacturing, Faculty of Engineering, The University of Nottingham, University Park, Nottingham, NG7 2RD United Kingdom; 2 School of Chemistry, The University of Nottingham, University Park, Nottingham, NG7 2RD United Kingdom; 3 Nanoscale and Microscale Research Centre, The University of Nottingham, University Park, Nottingham, NG7 2RD United Kingdom; 4 Department of Chemical and Environmental Engineering, Faculty of Engineering, The University of Nottingham, University Park, Nottingham, NG7 2RD United Kingdom

## Abstract

The fabrication of complex three-dimensional gold-containing nanocomposite structures by simultaneous two-photon polymerisation and photoreduction is demonstrated. Increased salt delivers reduced feature sizes down to line widths as small as 78 nm, a level of structural intricacy that represents a significant advance in fabrication complexity. The development of a general methodology to efficiently mix pentaerythritol triacrylate (PETA) with gold chloride hydrate (HAuCl_4_∙3H_2_O) is reported, where the gold salt concentration is adjustable on demand from zero to 20 wt%. For the first-time 7-Diethylamino-3-thenoylcoumarin (DETC) is used as the photoinitiator. Only 0.5 wt% of DETC was required to promote both polymerisation and photoreduction of up to 20 wt% of gold salt. This efficiency is the highest reported for Au-containing composite fabrication by two-photon lithography. Transmission Electron Microscopy (TEM) analysis confirmed the presence of small metallic nanoparticles (5.4 ± 1.4 nm for long axis / 3.7 ± 0.9 nm for short axis) embedded within the polymer matrix, whilst X-ray Photoelectron Spectroscopy (XPS) confirmed that they exist in the zero valent oxidation state. UV-vis spectroscopy defined that they exhibit the property of localised surface plasmon resonance (LSPR). The capability demonstrated in this study opens up new avenues for a range of applications, including plasmonics, metamaterials, flexible electronics and biosensors.

## Introduction

Metal-containing nanocomposites have numerous advanced optical, mechanical, electrical and photovoltaic properties^1^. Thus, recent advancement in the diverse fields of metamaterials, plasmonics, flexible electronics, biosensors, artificial implants and solar cells have generated a significant demand for the fabrication of Au-containing nanocomposites^2–4^. However, the traditional techniques for the production of such nanocomposites are either inherently limited to two-dimensional (2D) processing and/or involve multiple, time- and cost-intensive synthetic steps^3,5–8^. Two-photon lithography based additive manufacturing has been shown to overcome these limitations and fabricate arbitrary 3D micro/nano structures with resolution in the region of 100 nm^9–12^. Moreover, both polymeric and metallic structures have been manufactured respectively by two-photon induced polymerisation and metal salt reduction^13–18^, demonstrating the potential of the technology as a layer by layer manufacturing tool.

The combination of both processes into a single step to produce gold-containing nanocomposites was previously reported by Shukla *et al*.^19,20^, who demonstrated the fabrication of 2D periodic structures comprising *in situ* generated gold nanoparticles embedded in a matrix of the negative photoresist SU-8. Up to 10 wt% of gold salt was included in the formulation as a precursor for the resultant metallic nanoparticles. This method was further developed by Blasco *et al*. who created bridge-like conductive elements using an aqueous based solution containing 99.45 wt% of water in the resin mixture^21^. The fabrication of gold-containing pyramidal structures was shown to be possible by Liu *et al*. through the addition of a ruthenium-based dye to the resin mixture^22^. This enhanced the efficacy of the polymerisation process but the loadings were limited to 3 wt% of the gold salt. Whilst these studies clearly demonstrate the potential to create functional 3D nanocomposite structures, the need for a more efficacious way of inducing the formation of the matrix whilst controlling the growth of the nanoparticles is still a critically requirement. Further, for a range of applications, including for example the production of metamaterials and biosensors, it is desirable to be able control the gold concentration on demand. As a consequence it is important to develop a general formula which can support a broad range of gold concentrations and can be developed through two-photon lithography.

One barrier to the development of a general formula is the absence of a proven efficient photoinitiator that can be used to produce materials with a range of gold concentration. A candidate for this is 7-Diethylamino-3-thenoylcoumarin (DETC), which has been reported as an efficient photoinitiator for polymerisation^23–25^. Compared to commonly used Type I photoinitiators, such as Irgacure 819 and Irgacure 369, DETC can reduce the initiator requirement by up to 4 times (compared to Irgacure 819) or 14 times (compared to Irgacure 369)^23^. In fact, a level as low as 0.25 wt% DETC has been shown to be sufficient to achieve the polymerisation of pentaerythritol triacrylate (PETA) monomer^26^. However, no prior studies have reported the use of DETC as a photoinitiator for composite fabrication. This study reports the first demonstration of DETC as an efficient initiator for simultaneous two-photon polymerisation and photoreduction. In addition to promoting the polymerisation of PETA, only 0.5 wt% of DETC was sufficient to also promote the photoreduction of up to 20 wt% of the gold salt. This represents the highest efficiency for an initiator in the fabrication of gold-containing composites by two-photon lithography.

In this paper, the use of DETC as an efficacious photoinitiator to promote the formation of complex 3D gold-containing nanocomposite structures using simultaneous two-photon polymerisation and photoreduction is shown. It is also demonstrated that the concentration of the gold salt in the resin mixture can be readily varied between 0 and 20 wt%, offering potential for the control of the resultant hybrid microstructure. The feature size can be effectively reduced by incorporation of a gold salt into the resin mixture, with line width as small as 78 nm has been shown to be possible under these conditions. The structural intricacy demonstrated here represents a significant advance in the fabrication complexity achievable. The gold nanoparticles generated *in situ* exhibit a remarkable optical property of localised surface plasmon resonance (LSPR). The capability demonstrated in this study opens new avenues for a range of applications, including plasmonics, metamaterials, flexible electronics and biosensors. In particularly, user-defined 3D composite structures can be readily fabricated in this way.

## Results and Discussion

**Figure 1:**
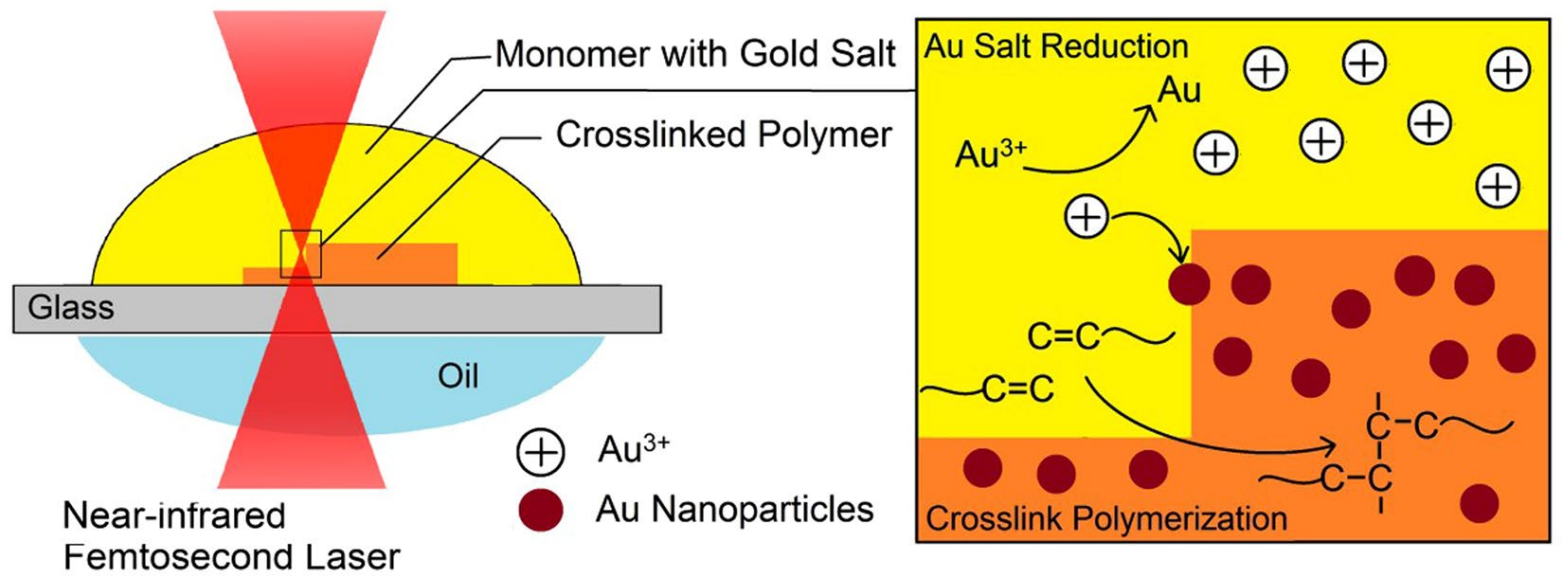
Illustration a general method of fabricating nanocomposites by simultaneous two-photon polymerisation and photoreduction. A drop of mixture (yellow) is loaded on a glass substrate which is composed of monomer, gold salt and photoinitiator. A near-infrared femtosecond laser beam (red) is focused into the mixture. A photoinitiator is excited by the simultaneous absorption of two photons, and triggers local chemical reactions, including monomer polymerisation, cross linking and metal salt reduction (image on the right). In this way, both polymer (orange) and metal nanoparticles (red) are formed simultaneously. Complex 3D structures are fabricated by scanning the laser in three dimensions.

A general additive manufacturing (AM) method to synthesis nanocomposites by simultaneous two-photon polymerisation and photoreduction is illustrated in Figure 1. The first pre-resin consists of PETA with 0.5–1.5 wt% of the photoinitiator DETC, whilst the second gold salt pre-resin contains gold(III) chloride hydrate (HAuCl_4_∙3H_2_O) and 0.5–1.5 wt% DETC dissolved in N,N-dimethylacetamide (DMAc). These two resins are always prepared to contain the same concentration of DETC which are then mixed to achieve the desired gold concentration. The compositions of the 15 individual formulations that were used in this study are summarized in Supplementary Table S1. A mixture composed of two pre-resins is loaded onto a glass substrate. A near-infrared (780 nm) femtosecond laser beam is then focused into the resin mixture, such that the photoinitiator is excited by the simultaneous absorption of two photons. This excitation subsequently triggered local chemical reactions, including monomer polymerisation, cross linking and metal salt reduction. In this way, both the polymer matrix and metal nanoparticles were formed simultaneously. As a direct result of this methodology, the *in-situ* generated nanoparticles were instantaneously embedded in a polymer matrix, preventing their further growth.

### Polymeric matrix formation

**Figure 2:**
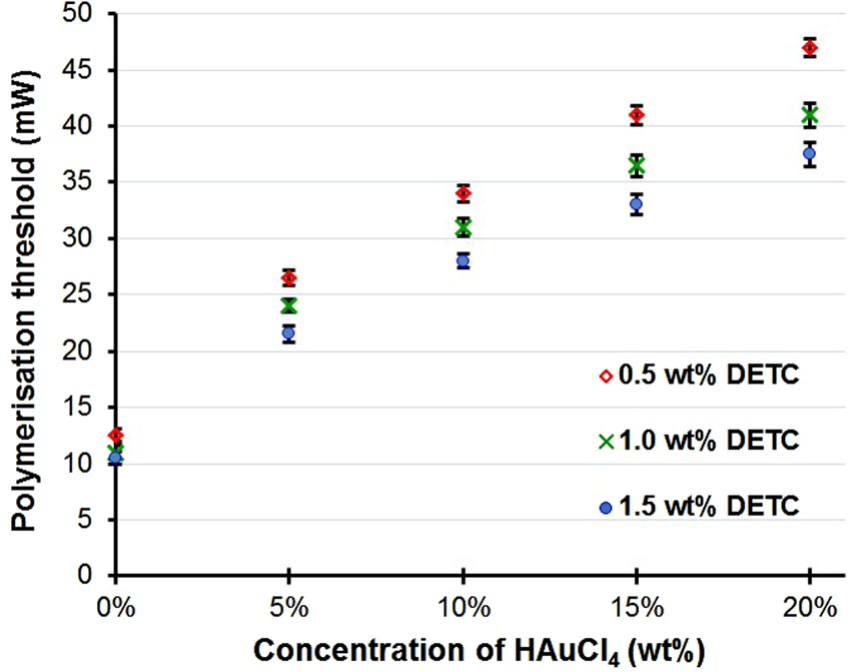
The relationship between the polymerisation threshold and the gold salt concentration for different levels of initiator concentration in the mixture. All the experiments were tested at a fixed scanning speed of 5000 μm/s.

The formation of simple structures using this two-photon methodology was conducted and confirmed using SEM. The DETC and the gold salt content was then systematically varied and the threshold (the minimum laser power) required for the formation of a structure with each gold loading was determined (See Supplementary Figure S3 for the details of the methodology). As shown in Figure 2, the presence of the gold salt significantly increased the laser energy required to achieve polymerisation. This extra energy is believed to be required to compensate for the energy consumption involved in the gold salt reduction.

For the polymerisation of pure PETA, levels of DETC as low as 0.25 wt% were demonstrated to be sufficient to achieve polymerisation. Further increases in the DETC concentration above 0.5 wt% were observed to have little effect on the polymerisation threshold. However, for the gold containing mixtures, our previous work on a Type I initiator in resin mixture had found that surplus initiator was needed, as both polymerisation and metal ion reduction consumed the generated free radicals. Thus a lack of free radicals was found to lower the efficacy of the reactions^27^. For example, it was shown that to fabricate PETA-Au composites containing 3 wt% gold precursor, 3 wt% Type I initiator 2-benzyl-2-(dimethylamino)-4′-morpholinobutyrophenone (DBMP) was required^22^. In this study, a Type II initiator (DETC) was found to be far more efficient in this process. Additional DETC was also noted to reduce the polymerisation threshold (see Figure 2). This was attributed to the higher concentration of initiator leading to a higher probability of radical formation, therefore the minimum required energy for radical formation was reduced accordingly.

For controlled 3D structure fabrication, sufficient material stability is needed to maintain the desired shape after laser exposure, *i.e*., the material must have sufficient rigidity imparted by the polymerisation process. In this study, the fabrication energy was typically set 10~20 mW above the threshold for this purpose. Furthermore, the laser power applied was also associated with the hatching distance (*the distance between adjacent lines of a fill pattern*), layer distance and geometry of the structure that can be achieved.

To confirm the success of the photopolymerisation process, samples were analysed by Raman spectroscopy (see Supplementary Figure S4 and Table S2). The degree of polymer conversion (DC) is estimated by measuring the ratio change of the peak areas associated with the C=C bonds to the C=O bonds before and after polymerisation. This is based on the assumption that during polymerisation and cross-linking, the C=C bonds in the monomer are attached by the radicals such that their band order is reduced and are converted to carbon-carbon single bonds (C‒C). Meanwhile, the C=O bonds do not change due to their non-participation in these radical reactions^22,28,29^. The DC of pure PETA was shown to be similar to the results reported by Jiang *et al*.^29^. Increases in the DC were observed with increasing laser power and the maximum achievable DC was limited to below 50% due to the restricted mobility of the oligomers. Our results also showed that under the same laser processing conditions, the DC decreases with a corresponding increase in the concentration of the gold precursor. This was associated with the energy consumption by the photoreduction, which leads to the effective energy for photopolymerisation being reduced accordingly. This finding is consistent with the observed relationship between the gold concentration and the polymerisation threshold. Furthermore, the presence of solvent molecules in the resin mixture was demonstrated to result in the effective dilution of the monomer, making the cross linking less efficient. Therefore, as the concentration of gold increases, higher laser energy is required.

**Figure 3:**
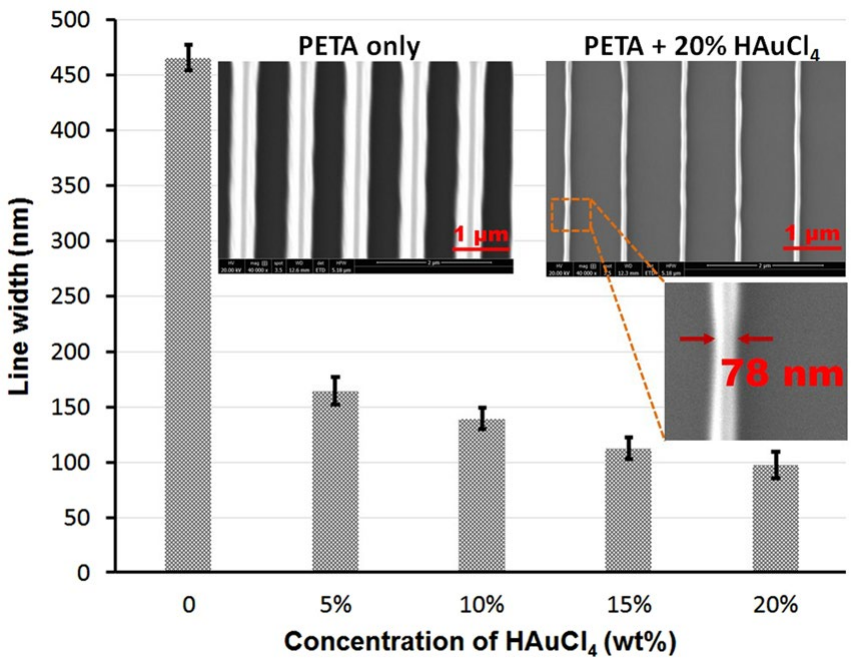
The relationship between the line width and the gold salt concentration in the resin mixture. Five samples (with 0%, 5%, 10%, 15% and 20% of gold salt) were prepared under the same processing conditions. A group of lines were fabricated using laser power of 50 mW and scanning speed of 5000 µm/s. The SEM image on the left shows the sample without gold. The average line width is 466 nm. The SEM image on the right shows the sample prepared with 20% gold salt present in the resin mixture. The average line width is 97 nm. Both images were taken with 40,000x magnification. The inserted image shows the minimum achievable line width is 78 nm.

It was also found that the feature size of the fabricated structure that could be achieved decreased with increasing gold salt concentration in the mixture. Figure 3 compares five samples prepared under the same processing conditions. The line width of the sample prepared with 20% gold salt was about 4.8 times smaller than that of the sample without gold salt, exhibiting an average line width of 97 nm and minimum line width of 78 nm. This phenomenon of feature size reduction was also observed in fabricated 3D structures. The reduced feature size was mainly attributed to the energy consumed by the gold salt reduction lowering the effective energy available for polymerisation and so narrowing the portion of the laser beam which is above the threshold^22^. This indicates that the gold salt can be used as a further lever to reduce feature size and control the fabrication resolution achieved during two-photon lithography.

### Photoreduction

**Figure 4:**
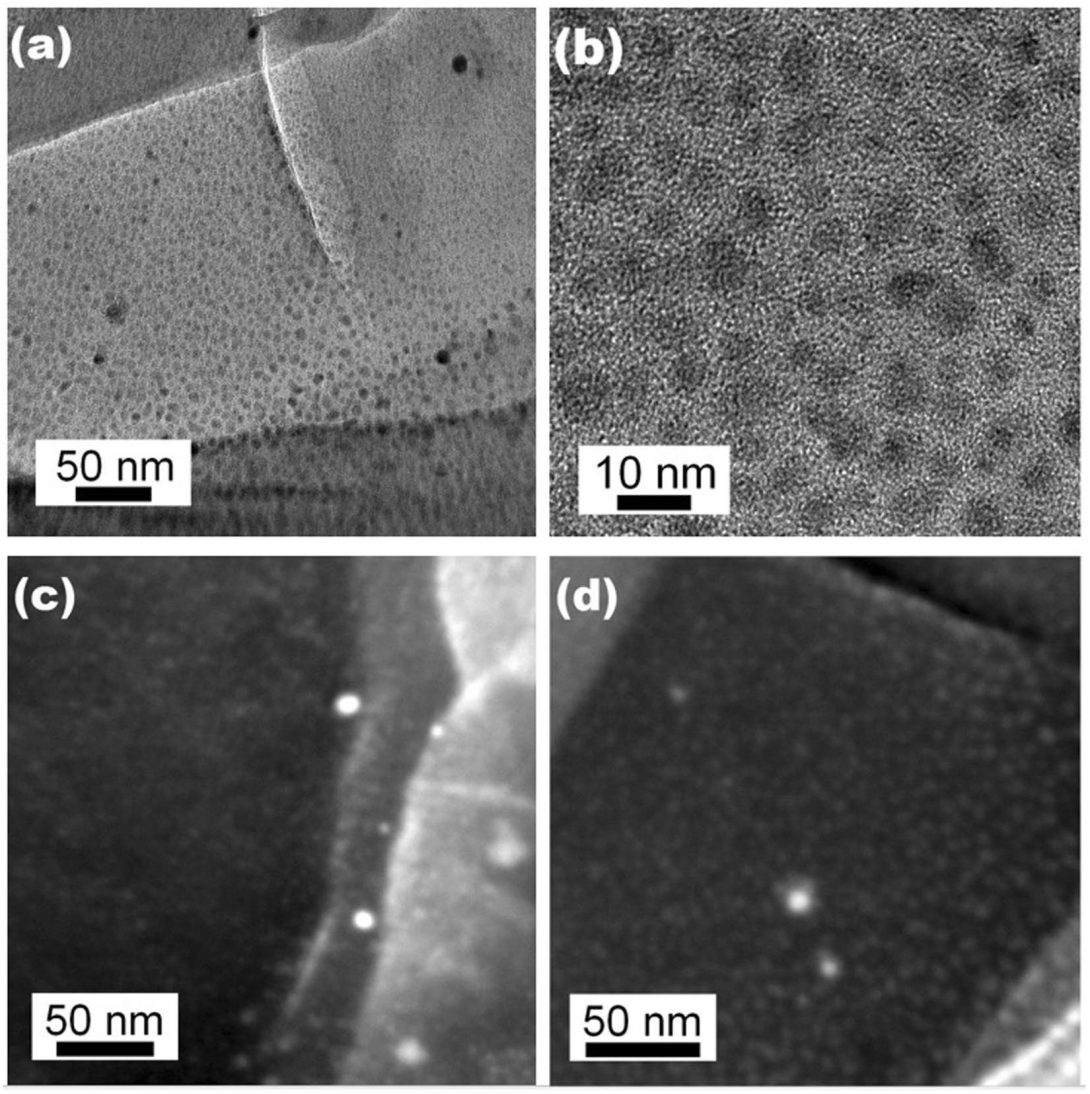
(**a**,**b**) TEM images and (**c**,**d**) Dark Field Scanning Transmission Electron Microscopy (DF-STEM) images showing the presence of small Au nanoparticles (5.4 ± 1.4 nm for long axis and 3.7 ± 0.9 nm for short axis) embedded in polymer matrix and some large particles located on the surface. The TEM images were recorded in bright field, where species with high atomic number appear as dark features. The DF-STEM images were recorded in dark field, where species in high atomic contract appear as bright features. The analysed sample was prepared using Formulation 15 (PETA-20% Au-1.5% DETC).

The successful reduction of gold salt to form gold nanoparticles was confirmed by both TEM and Dark Field Scanning Transmission Electron Microscopy (DF-STEM) analysis, as shown in Figure 4 and Supplementary Figure S5. Elliptical particles were noted to be uniformly distributed within the polymer matrix. The average sizes of the particles were 5.4 ± 1.4 nm for long axis and 3.7 ± 0.9 nm for short axis (please refer Supplementary Figures S7 and S8 for statistical analysis). Some large nanoparticles (>10 nm) were also found on the top surface. The formation of these large particles could be associated with the aggregation of small particles at the surface, where their growth is unconstrained by the presence of the polymer matrix. EDX analysis confirmed they were all Au-containing particles. TEM images were also captured by directly analysing the structure made by two-photon fabrication. To achieve this a helix structure was fabricated and attached to the end of a glass substrate. A small piece was later transferred to a TEM grid using a micro-manipulator (see Supplementary Figure S6). By choosing suitable laser power and scan speed, it was then possible to fabricate the structure sufficiently thin for TEM analysis.

**Figure 5:**
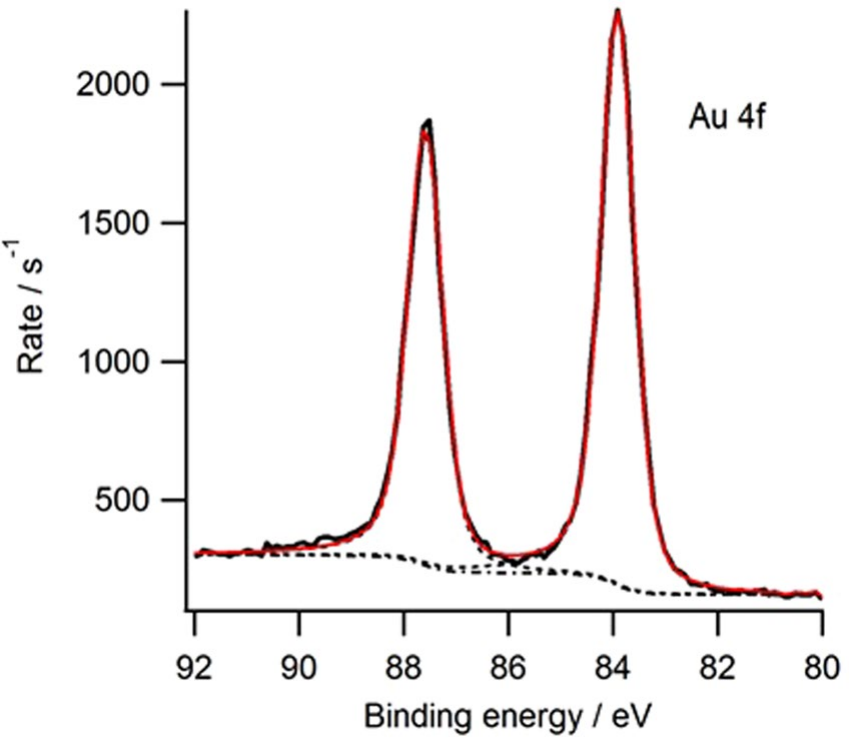
The Au 4f range of XPS spectrum of PETA-Au composite. The analysed sample was prepared using Formulation 7 (PETA-5% Au-1% DETC).

The successful reduction to the energy states associated with elemental gold was further confirmed by XPS analysis. A low resolution wide scan XP spectrum over the full energy range was shown in Supplementary Figure S9. Since no Cl was observed in the wide scan spectrum, it is reasonable to assume that no gold chlorides from the starting material were present. The reduction from Au^3+^ to Au^0^ involves several linked reactions, which also normally generate two intermediate states - Au^2+^ and Au^1+^^20,22,30–33^. The Au 4f was of particular interest as it would indicate the oxidation state of the Au. Figure 5 shows an Au 4f high resolution XP spectrum (black solid line), with a peak fit based on a Shirley background and two asymmetric lf (0.9, 1, 200, 300) components modelling the spin orbit splitting of the Au 4f_7/2,5/2_ doublet (dotted black lines), and the model envelope is shown in red. The FWHM of these was 0.77 and 0.79 eV respectively. The Au 4f_7/2_ component was measured at a B.E. of 83.9 eV. There was no obvious shoulder to high B.E. which would appear if the gold was oxidised. Our own reference bulk gold material produced values of FWHM 0.64 & 0.65 eV, B.E. = 84.1 eV. The simple asymmetric peak fit indicates that the Au is likely to be purely metallic form, and the slight broadening of the peaks compared to bulk gold is likely to be due to the nanoparticle size.

### DETC as Photoinitiator

Table 1 listed the various initiators used to fabricate the two-photon based Au-containing composites, where both weight and molar ratios were calculated for the given formulation. Comparison for the initiator efficiency was based on the weight ratio/molar ratio of gold salt over initiator in the resin, and by assuming the gold salt was completely reduced by the initiator present. In the current study, it was demonstrated that as low as 0.5 wt% DETC can reduce 20 wt% gold salt (Formulation 5), *i.e*., on a wt%:wt% basis, 1 unit of initiator DETC was found to reduce 40 units of gold salt, in addition to promoting polymerisation. This efficiency is higher than that of the alternative initiators used in previously published work. For example, previous work used Type I initiator DBMP and 3 wt% initiator can only promote the photoreduction of up to 3 wt% of the gold salt, in addition to promoting the polymerisation of PETA^22^. Extra DBMP was needed for reducing higher concentrations of gold salt in the resin mixture.

**Table 1:** Compare initiator used for Au composite fabrication by two-photon techniques.

Monomer	Initiator	Formulation	Gold salt: initiator		Reference
			Weight ratio	Molar ratio	
SU-8	PC2506 (photoacid generator in SU-8) + AF380	10 wt% gold salt, 1 wt% AF380, 1–5 wt% PC2506	<5:1	N/A	^19,20^
Acrylate-functionalized poly(ethylene glycol) derivative	Irgacure 2959	0.2 wt% gold salt, 0.15 wt% Irgacure 2959	4:3	1:1	^21^
TMPTA, PETA	DBMP + Ru(II) complex	3 wt% gold salt, 3 wt% DBMP, 0.1 wt% Ru(II) complex	1:1	1:1	^22^
PETA	DETC	20 wt% gold salt, 0.5 wt% DETC (Formulation 5)	40:1	39:1	This paper

*PC2506: a diaryliodonium salt photoacid generator, Ar_2_I^+^SbF_6_^−^; AF380: an initiator developed by the US Air Force Research Laboratory with unknown chemical structure; Irgacure 2959: (1-[4-(2-hydroxyethoxy)-phenyl]-2-hydroxy-2-methyl-1-propane-1-one); TMPTA: trimethylopropane triacrylate; DBMP: 2-benzyl-2-(dimethylamino)-4′-morpholinobutyrophenone; Ru(II) complex: tris(2,2′-bipyridyl) dichlororuthenium(II) hexahydrate.

**According to the Safety Data Sheet of SU-8 2000 series, the concentration of initiator in commercial SU-8 is 1–5%.

Some indication of why this might occur can be obtained from recent descriptions of the radical generation process for DETC^23,34,35^. It is understood that this occurs through an excited state absorption process, which is different from most commonly used initiators, e.g. Irgacure 369 and Irgacure 819, where radical generation occurs through a homolytic C-C bond scission^35^. Therefore, it is proposed that it is this excited state absorption process that leads to high efficiency when producing Au based composites by the methods described in this paper.

### 3D Composite Structures

**Figure 6:**
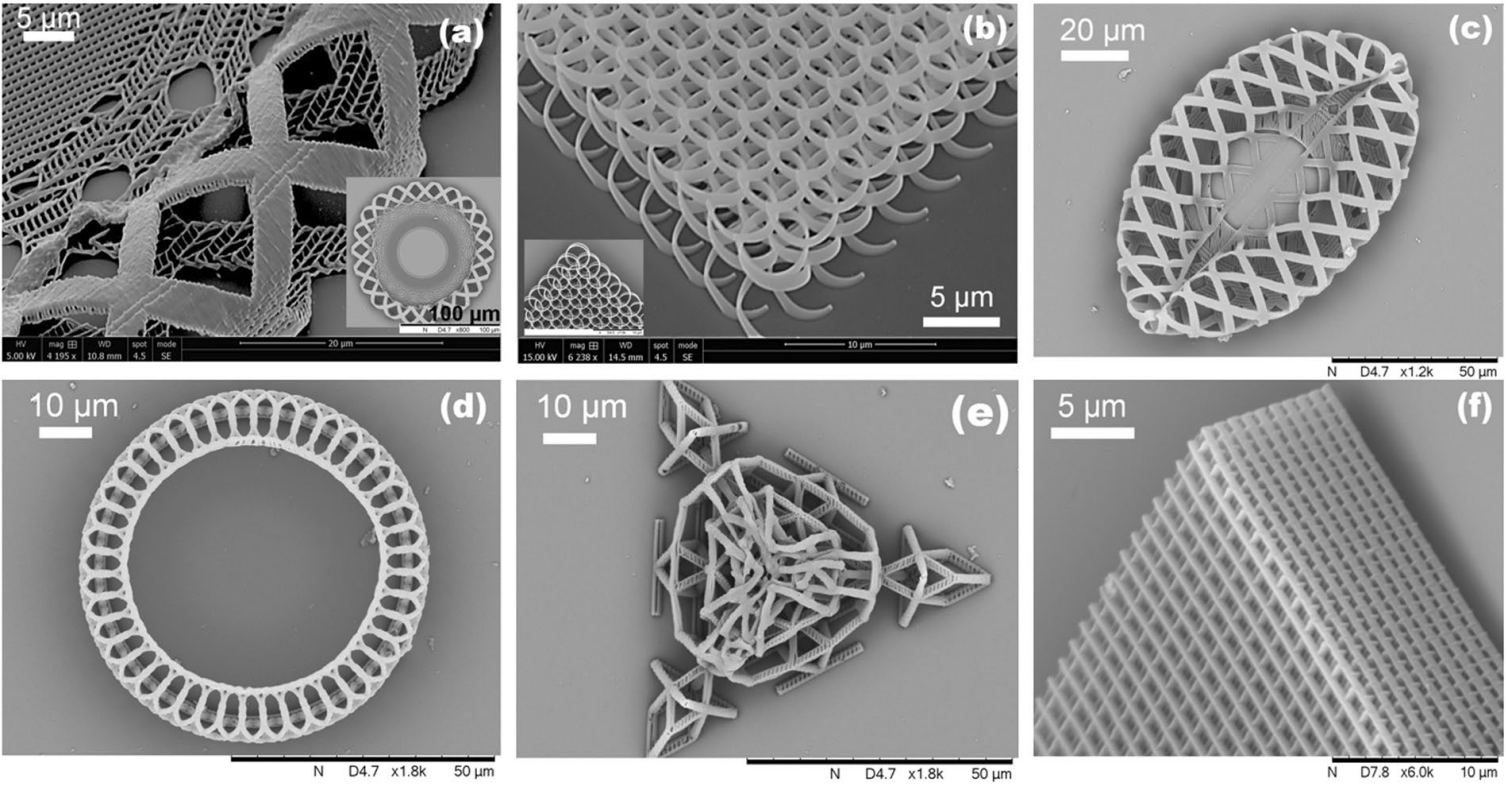
SEM images showing complex 3D Au-containing composite structures fabricated by simultaneous two-photon polymerisation and photoreduction.

A beneficial consequence of the reliable and efficient simultaneous two-photon polymerisation and photoreduction achieved was that Au-containing complex 3D structures could be fabricated. To demonstrate this fabrication capability, a number of geometrically complex structures were manufactured. Figure 6 shows a two ring structure (a) & (d), an inter-linked helix structure (b), an ellipse structure (c), a pyramid structure (e), and a woodpile photonic crystal structure (f). Please refer Supplementary Figure S10 for the magnified tilted images of the ring structure and the pyramid structure shown in Figure 6(d) and (e).

The fabrication capability in achieving complex 3D structures demonstrated in the current work is far in advance of previously published work. Shukla *et al*. showed the capability of fabricating 2D structures by combining two-photon polymerisation and photoreduction^19,20^. The capability of fabricating 3D structure was first demonstrated by Blasco *et al*., however, only simple bridge-like elements have been shown^21^. Liu *et al*. demonstrated the capability to fabricate a pyramid-like solid structure^22^. Here in this paper we have demonstrated that both complex and free standing objects can be made.

Local burning was sometimes observed, which was attributed to plasmonic heating of the *in situ* generated Au nanoparticles^36,37^. This effect was particularly prevalent when the gold salt concentration exceeds 10 wt%. This demonstrated that the level of laser power and scan speed applied in different formulations needed to be carefully selected to minimize this effect.

### Future applications

**Figure 7:**
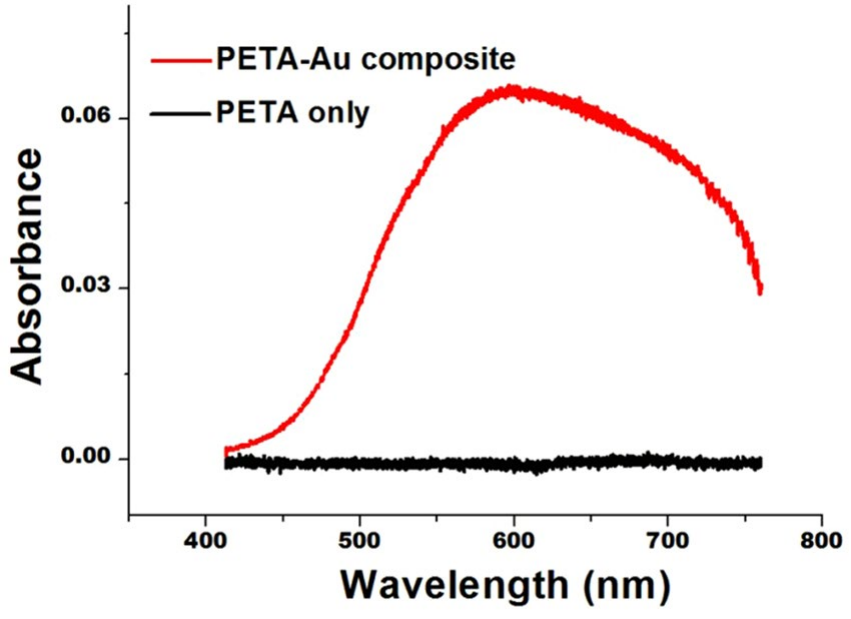
UV-vis spectra of PETA sample (without Au) (black) and PETA-Au composite sample (red). The analysed sample was prepared using Formulation 11 (PETA-1.5% DETC) and Formulation 15 (PETA-20% Au-1.5% DETC).

The *in situ* generated gold nanoparticles were found to exhibit the unusual optical property of the localised surface plasmon resonance (LSPR) absorbance^38^, a collective dipole oscillation of the 6 s conduction electrons of the surface gold atoms in a nanoscale object that are coherent with the incoming electromagnetic radiation. This gives rise to a broad absorbance band in the visible part of the spectrum, as shown in Figure 7. By comparison, the pure PETA sample is absent of any absorbance features within the analysed energy range. It is well known that the size, shape, dispersity and dielectric environment surrounding nanoscale gold particles have a significant effect on the position, symmetry and intensity of the LSPR absorbance, with ~5 nm gold nanoparticles often appearing as wine red coloured solutions (with a maximum absorbance centred at ~525 nm). However, the significant redshift of the mean position of the LSPR band to ~600 nm and the presence of a shoulder above 700 nm are strongly indicative of the formation of one-, two- and three-dimensional aggregated networks of nanoparticles, often observed in solution and in polymer matrices^39,40^. The position of the absorption band has been reported to shift to higher wavelength with increasing laser power^39^. Please note for current measurement, PETA-Au composite thin film with an area of 1.5 mm × 1.5 mm were prepared, using Formulation 15 which contained 20% gold salt. To fabricate such a large structure with a high concentration of gold, large Au particles (>100 nm) are sometimes observed on substrate, which may result in the absorption band demonstrating this drift to higher wavelength. The observed LSPR absorbance in Au-containing composites fabricated by two-photon lithography opens new avenues for a range of applications, including plasmonics, metamaterials, flexible electronics, catalysis and biosensors^38,41–43^. In particular, user-defined 3D composite structures can be readily fabricated in this way. The reduced feature size assisted by gold salt in the resin has pushed the length scale of the structures close to the nanoparticles themselves.

## Conclusions

A general formula for combining PETA with gold chloride hydrate to fabricate complex 3D Au-containing nanocomposites by simultaneous two-photon polymerisation and photoreduction has been demonstrated. Complex 3D structures could be successfully generated when the concentration of the gold salt in the resin mixture was in a range from zero up to 20 wt%. For the first-time DETC has been used as a photoinitiator to achieve simultaneous two-photon polymerisation and photoreduction. Furthermore, it has been identified to be the most efficient initiator reported to date, where 1 unit mass of DETC can also promote the reduction of up to 40 unit mass of gold salt, in addition to promoting polymerisation. This efficiency was attributed to the excited state absorption of radical generation. Both the polymerisation and gold salt reduction reactions have been verified by a range of microscopy and spectroscopy approaches. The polymerisation threshold was shown to increase with increasing gold salt concentration and decrease with increasing initiator concentration, which was linked to the overall level of energy needed to generate the level of radical required to complete both chemical transformations. For a particular radical concentration its degree of polymer conversion was observed to decrease with the increasing gold salt concentration which again was related to radical use in the reduction. The feature size can be tuned by the addition of the gold salt to the resin mixture, with the ability to produce line widths as small as 78 nm demonstrated, which is linked to a smaller portion of the laser beam would being able to exceed its target threshold value. Gold particles of well controlled size, *i.e*., average size of 5.4 ± 1.4 nm for the long axis and 3.7 ± 0.9 nm for the short axis, were shown to be generated due to being growth restricted by the polymer matrix, and a few large particles can be found on surface whose size had not been controlled. All the *in-situ* generated gold nanoparticles exhibit a characteristic optical property of localised surface plasmon resonance. The manufacture of various complex 3D structures has been demonstrated, where the complexity demonstrated here is far in advance of those previously observed. The technology demonstrated in this paper represents a new opportunity to develop functional devices suitable for various applications, such as plasmonics, metamaterials, flexible electronics and biosensors.

## Methods

### Materials

Monomer PETA, gold salt HAuCl_4_∙3H_2_O, and solvent DMAc were sourced from Sigma-Aldrich, UK. Initiator DETC was sourced from Angene, UK. Due to the limited solubility of DETC in PETA (<2 wt%), mixtures of 0.5 wt%, 1 wt% and 1.5 wt% DETC were investigated. The chemical structures of PETA and DETC are illustrated in Figure S1. The optical absorption spectra of pure DMAc, PETA in DMAc, DETC in DMAc and gold chloride hydrate in DMAc are shown in Figure S2. In all spectra, no absorption band is observed at the laser wavelength of 780 nm, which implies that the photo-induced reactions were associated with exciting the chemicals by the two-photon absorption process.

### Nanocomposite fabrication

Au-containing nanocomposites were fabricated using a commercial two-photon lithography system – Nanoscribe Photonic Professional GT, equipped with a fiber laser at a wavelength of 780 nm, pulse frequency of 80 MHz and pulse duration of 120 fs. An oil immersion objective (63x, NA = 1.4, WD = 190 µm) was used to focus the laser beam. Micro/nano 3D structures were formed by moving the laser beam in X-Y directions using a galvo-scanner and moving a piezo stage in the Z direction accordingly, or fixing the focus of the laser beam and moving a piezo stage in X, Y and Z directions accordingly. The laser power used was in the range of 10 mW to 60 mW. The scanning speed was varied from 100 μm/s up to 5000 μm/s.

Composite fabrication was carried out by first loading a drop of the resin mixture onto a clean cover slip. Then the cover slip was put on the stage of Nanoscribe for two-photon processing. Following laser exposure, the sample was developed by first soaking in propylene glycol monomethyl ether acetate (PGMEA) (Sigma-Aldrich, UK) for 15 min then in 2-propanol (Sigma-Aldrich, UK) for 2 min to remove unreduced gold salt and residual monomer. Finally, the sample was dried by nitrogen gas. Both the resin preparation and composite fabrication were carried out in UV-free environment. All the chemicals used in current study were used as received.

### Characterization

Two-photon polymerisation was verified by checking the surface morphology by scanning electron microscopy (SEM) (Hitachi TM3030 and FEI Quanta 650) and chemical analysing by Raman Spectroscopy (Horiba–Jobin–Yvon LabRAM). Two-photon induced gold salt reduction was verified by checking the surface morphology by Transmission Electron Microscopy (TEM) (JEOL 2100+), and chemical analysing by Energy Dispersive X-ray Spectroscopy (EDX) (Oxford Instruments X-MaxN 80) and X-ray Photoelectron Spectroscopy (XPS) (Kratos Analytical Ultra-2008). The optical absorbance of fabricated structures was measured using UV-vis spectroscopy (see Supplementary information for detail).

The datasets generated during and/or analysed during the current study are available from the corresponding author on reasonable request.

## Electronic supplementary material

Supplementary information
