# Author Correction: Additive manufacture of complex 3D Au-containing nanocomposites by simultaneous two-photon polymerisation and photoreduction

**DOI:** 10.1038/s41598-018-21513-8

**Published:** 2018-02-19

**Authors:** Qin Hu, Xue-Zhong Sun, Christopher D. J. Parmenter, Michael W. Fay, Emily F. Smith, Graham A. Rance, Yinfeng He, Fan Zhang, Yaan Liu, Derek Irvine, Christopher Tuck, Richard Hague, Ricky Wildman

**Affiliations:** 1 Centre for Additive Manufacturing, Faculty of Engineering, The University of Nottingham, University Park, Nottingham, NG7 2RD United Kingdom; 2 School of Chemistry, The University of Nottingham, University Park, Nottingham, NG7 2RD United Kingdom; 3 Nanoscale and Microscale Research Centre, The University of Nottingham, University Park, Nottingham, NG7 2RD United Kingdom; 4 Department of Chemical and Environmental Engineering, Faculty of Engineering, The University of Nottingham, University Park, Nottingham, NG7 2RD United Kingdom

Correction to: *Scientific Reports* 10.1038/s41598-017-17391-1, published online 07 December 2017

The Acknowledgements section in this Article is incorrect.

“This work was undertaken at the University of Nottingham funded by the Engineering and Physical Sciences Research Council (EPSRC) through the grants EP/I033335/2 “EPSRC Centre for Innovative Manufacturing in Additive Manufacturing” and EP/N024818/1 “Formulation for 3D Printing”, and by the Air Force Office of Scientific Research (AFOSR) through award FA9550-14-1-0048. ES wants to acknowledge the EPSRC grant for the Kratos LiPPS XPS instrument EP/K005138/1.”

should read:

“This work was supported by the Engineering and Physical Sciences Research Council [grant number EP/I033335/2, EP/N024818/1, EP/K005138/1]; the Air Force Office of Scientific Research (AFOSR) [grant number FA9550-14-1-0048].”

