# Supplementary material for: Additive manufacture of complex 3D Au-containing nanocomposites by simultaneous two-photon polymerisation and photoreduction
Source: Sci Rep. 2017 Dec 7;7:17150. doi: 10.1038/s41598-017-17391-1 (PMC5719407; doi:10.1038/s41598-017-17391-1)
Supplement: Supplementary file 1 — Supplementary information [file 41598_2017_17391_MOESM1_ESM.pdf]

Supplementary information

Additive manufacture of complex 3D Au-containing nanocomposites by simultaneous two-photon polymerisation and photoreduction

Qin Hu,[1*] Xue-Zhong Sun,[2] Christopher D. J. Parmenter,[3] Michael W. Fay,[3] Emily F. Smith,[3] Graham A. Rance,[3] Yinfeng He,[1] Fan Zhang,[1] Yaan Liu,[1] Derek Irvine,[4] Christopher Tuck,[1] Richard Hague[1] and Ricky Wildman[1*]

[1] Centre for Additive Manufacturing, Faculty of Engineering, The University of Nottingham, University Park, Nottingham, United Kingdom NG7 2RD

[2] School of Chemistry, The University of Nottingham, University Park, Nottingham, United Kingdom NG7 2RD

[3] Nanoscale and Microscale Research Centre, The University of Nottingham, University Park, Nottingham, United Kingdom NG7 2RD

[4] Department of Chemical and Environmental Engineering, Faculty of Engineering, The University of Nottingham, University Park, Nottingham, United Kingdom NG7 2RD

[*] Email: ricky.wildman@nottingham.ac.uk; qin.hu@nottingham.ac.uk

Materials

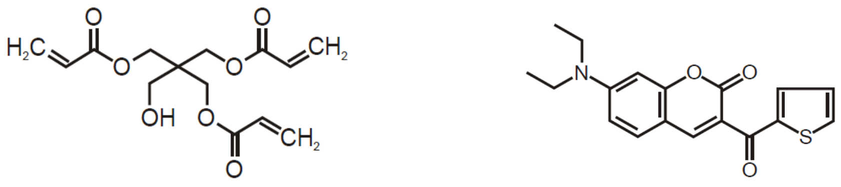

(a) (b)

Figure S1: Chemical structure of (a) monomer PETA and (b) photoinitiator DETC.

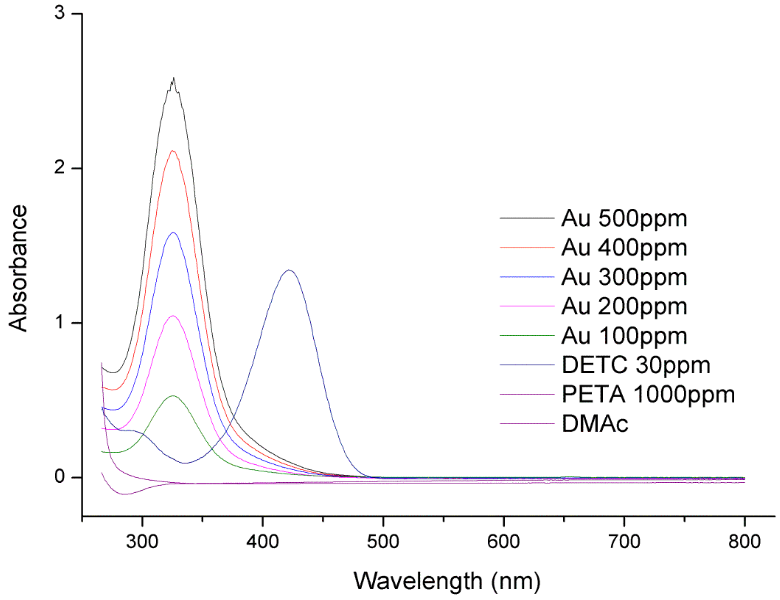

Figure S2: The optical absorption spectra of pure DMAc, PETA in DMAc, DETC in DMAc and tetrachloroauric acid trihydrate in DMAc at different concentrations. In all spectra, no absorption band is observed at the laser wavelength of 780 nm, which implies that the photo-induced reactions were associated with exciting the chemicals by the two-photon absorption process.

Table S1: Composition of 15 formulations

Formulation		Resin A	Resin B	Mix Resin A with Resin B*	
				HAuCl$_4$ (wt%)	DETC (wt%)
1	PETA-0.5%DETC	99.5%PETA + 0.5%DETC	49.75% HAuCl$_4$ + 49.75% DMAc + 0.5% DETC	0	0.5
2	PETA-5%Au-0.5%DETC			5	
3	PETA-10%Au-0.5%DETC			10	
4	PETA-15%Au-0.5%DETC			15	
5	PETA-20%Au-0.5%DETC			20	
6	PETA-1%DETC	99% PETA + 1% DETC	49.5% HAuCl$_4$ + 49.5% DMAc + 1% DETC	0	1
7	PETA-5%Au-1%DETC			5	
8	PETA-10%Au-1%DETC			10	
9	PETA-15%Au-1%DETC			15	
10	PETA-20%Au-1%DETC			20	
11	PETA-1.5%DETC	98.5% PETA + 1.5% DETC	49.25% HAuCl$_4$ + 49.25% DMAc + 1.5% DETC	0	1.5
12	PETA-5%Au-1.5%DETC			5	
13	PETA-10%Au-1.5%DETC			10	
14	PETA-15%Au-1.5%DETC			15	
15	PETA-20%Au-1.5%DETC			20	

* As DMAc is used as a solvent and plays no role in polymerisation and photoreduction, the concentration of gold talked in this paper is calculated by the weight ratio of gold chloride hydrate vs the total weight of gold chloride hydrate, PETA and DETC.

Polymerisation

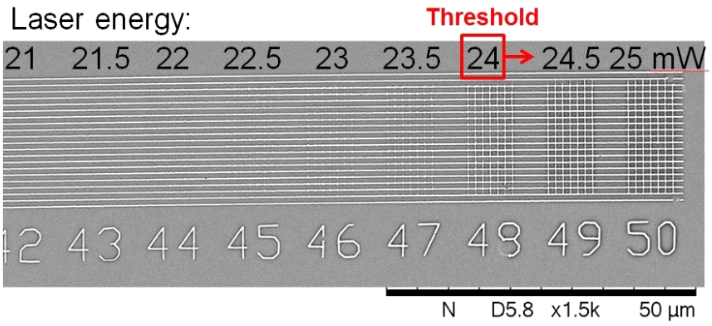

Figure S3: SEM image showing how the polymerisation threshold was defined according to the integrity of the lines. Please note the number written under each group of testing lines represents the percentage of laser power specified by Nanoscribe. The actual laser energy used is that number times 50 mW. In the sample shown here, the threshold is 48% x 50 mW = 24 mW. To minimize the error caused by interface finding (*if a laser beam is focused beneath the substrate due to uneven surface, although the power is high enough for polymerisation, no polymeric lines can be drawn on substrate*), a series of horizontal lines were printed beforehand using a fixed laser power. They acted as a supporting layer to ensure a level base above the substrate. The lines for testing were then fabricated on top of the horizontal lines in a direction rotated 90° relative to the first lines, and at various laser powers.

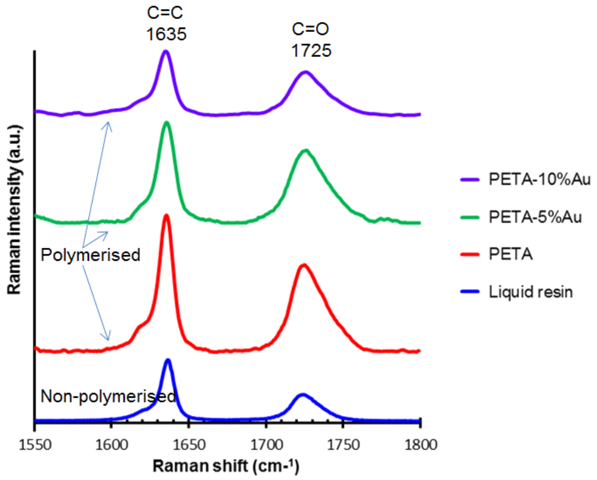

Figure S4: Raman spectra of (i) non-polymerised monomer (blue),(ii) PETA without Au, polymerised Formulation 11 (red), (iii) PETA with 5 wt% gold salt, polymerised Formulation 12 (green) and (iv) PETA with 10 wt% gold salt, polymerized Formulation 13 (purple) in the range 1550-1800 cm^1. The spectra have been baseline-corrected and shifted on the vertical axis for viewing clarity. The polymerised samples were prepared under the same processing conditions (laser power of 42.5 mW, scanning speed of 5000 µm/s, hatching distance of 0.8 µm) to afford woodpile structures with 4 layers. Two distinctive peaks at 1635 and 1725 cm^{-1} are observed in all cases, associated with the carbon-carbon double bonds (C=C) and the carbon-oxygen double bonds (C=O) respectively [1]. During cross-linking, the bond order of the C=C bonds adjacent to the ester groups in the monomer is reduced as a consequence of the

Michael addition of monomer units, yielding single C-C bonds and a corresponding reduction in the intensity of the vibrational mode associated with C=C bonds at 1635 cm^{-1} However, the C=O bonds do not participate in these reactions and thus no change in their intensity at 1725 cm^{-1} is observed. Therefore, by measuring the change in the intensity (as peak areas) ratio of the C=C and C=O bonds before and after polymerisation, the degree of polymer conversion (DC) can be estimated [1-3].

Table S2: Degree of polymer conversion of polymerized PETA and PETA-Au composites

**Sample**<br>Woodpile structure with 4 layers<br>(Laser power = 42.5 mW,<br>Scanning speed = 5000 µm/s,<br>Hatching distance = 0.8 µm,<br>Initiator concentration = 1.5 wt%)	**Degree of polymer conversion (DC):**<br>$DC = 1 - \frac{A_{C=C} / A_{C=O}}{A'_{C=C} / A'_{C=O}}$<br>where, , and are the peak intensities in the Raman spectra related to the C═C and C═O groups in the polymerised structures and the non-polymerised liquid resin.
PETA	42%
PETA + 5%$HAuCl_4$	35%
PETA + 10%$HAuCl_4$	27%

As noted by Burmeister et al [2], the hatching distances also influence the DC of the pure polymer, with the DC increasing with a corresponding reduction in the hatching distance. This relationship has been attributed to the effective increase in total exposure time and the influence of created radicals to the adjacent rods [2]. Here for composite fabrication, this relationship still applies. In addition, the presence of *in situ* generated Au nanoparticles can absorb laser energy and on occasions facilitate local burning through plasmonic heating effects. To minimise the influence of these factors on the DC, the samples shown in Figure S4 were prepared with a hatching distance of 0.8 µm, and adjacent rods were fabricated with a pause of 1 second interval. For general 3D feature fabrication, the hatching distance is normally less than 0.8 µm and without pause or with shorter pause between adjacent lines or layers. Then the actual achievable DC can be higher.

TEM analysis

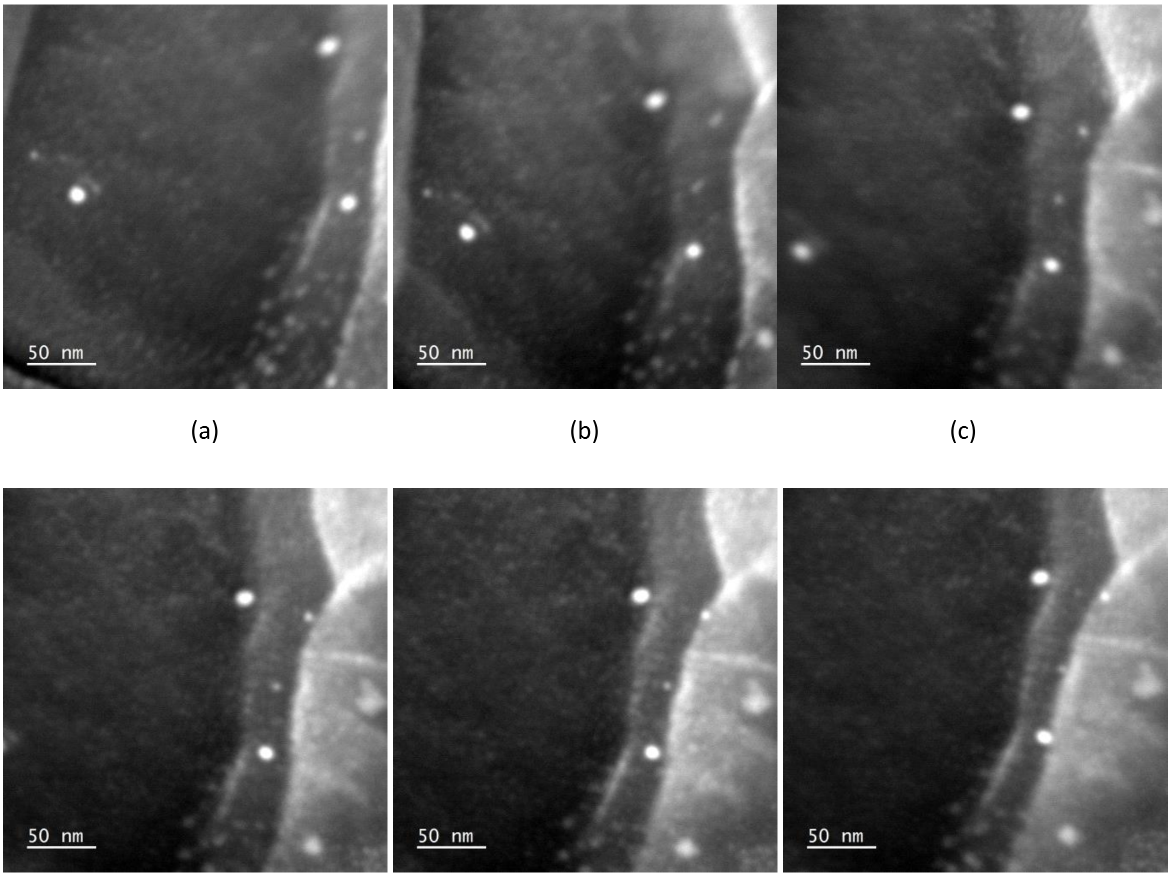

(a) (b) (c)

(d) (e) (f)

Figure S5: A series of Dark Field Scanning Transmission Electron Microscopy images taken at different tilts showing the distribution of particles throughout the thickness of the layer.

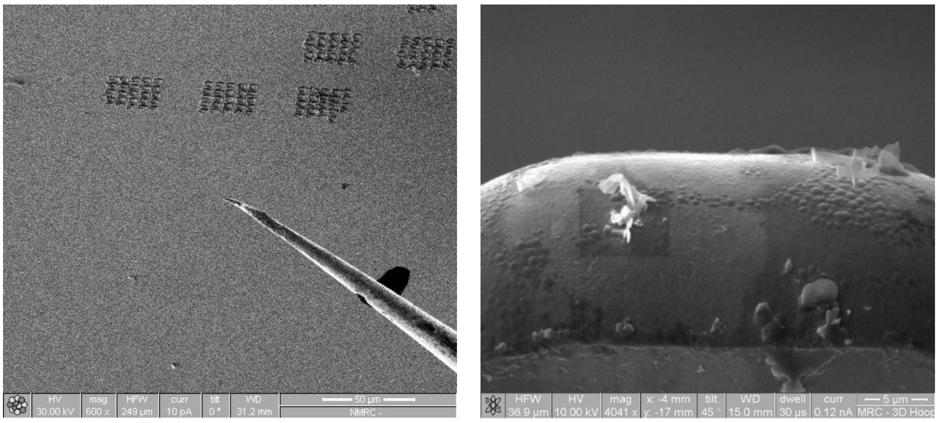

(a) (b)

Figure S6: SEM images illustrate how to prepare TEM sample. (a) A piece of helix structure prepared by simultaneous two-photon polymerisation and photoreduction was picked up by a micro-manipulator; and (b) was then transferred to a TEM sample holder.

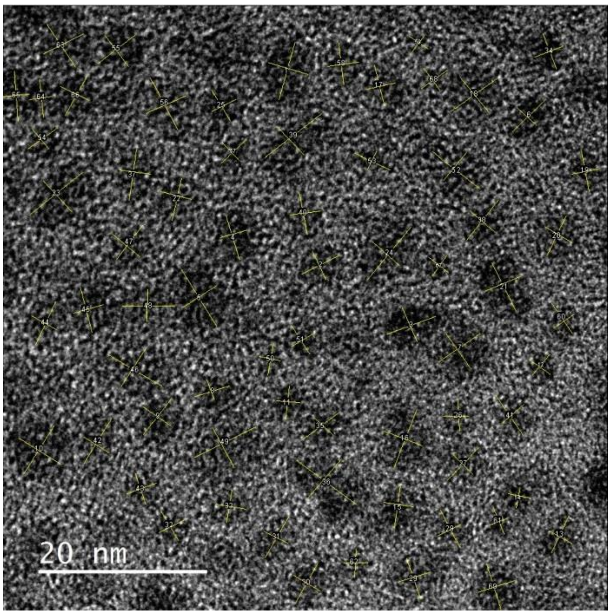

Figure S7: Illustration of 69 particles picked from TEM image for size measurement.

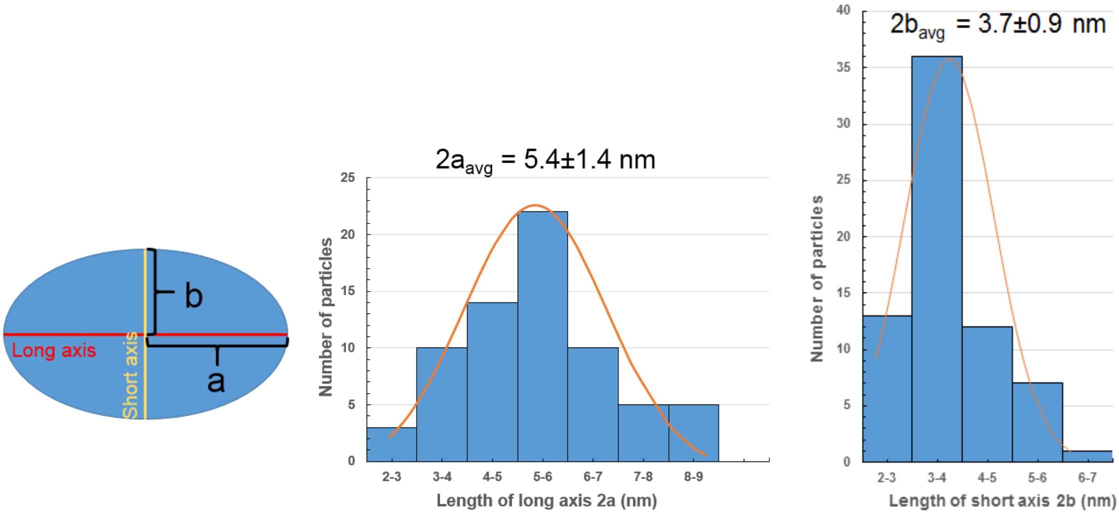

(a) (b) (c)

Figure S8: (a) Illustration of the long axis and the short axis of an elliptical particle. (b) –(c) Particle size distribution. The orange curve shows the fitted normal distribution. The average sizes of the particles are 5.4±1.4 nm for long axis and 3.7±0.9 nm for short axis.

XPS analysis

Samples were analysed using the Kratos AXIS ULTRA with a mono-chromated Al kα X-ray source (1486.6eV) operated at 10 mA emission current and 12 kV anode potential (120 W). Samples were mounted on a standard Kratos sample bar using double sided tape and pumped down to UHV overnight before insertion to the analysis chamber. A charge neutralizer filament was used to prevent surface charging. Hybrid–slot mode was used measuring a

sample area of approximately 0.5 mm^2, note that this is significantly larger than the size of the structure being analysed so a large proportion of the signal detected in the wide scan will come from the surrounding glass substrate material. The analysis chamber pressure was better than 5 x 10^{-9} mbar. A wide scan at low resolution (1400 - -5 eV binding energy range, pass energy 80 eV, step 0.5 eV, sweep time 20 minutes). High resolution spectra at pass energy 20 eV with step of 0.1 eV, sweep times of 10 minutes each were also acquired for photoelectron peaks from the detected elements. The high resolution spectra were charge corrected to the C 1s peak set to 285 eV. Casaxps (version 2.3.18dev1.0x) software was used for quantification and spectral modelling.

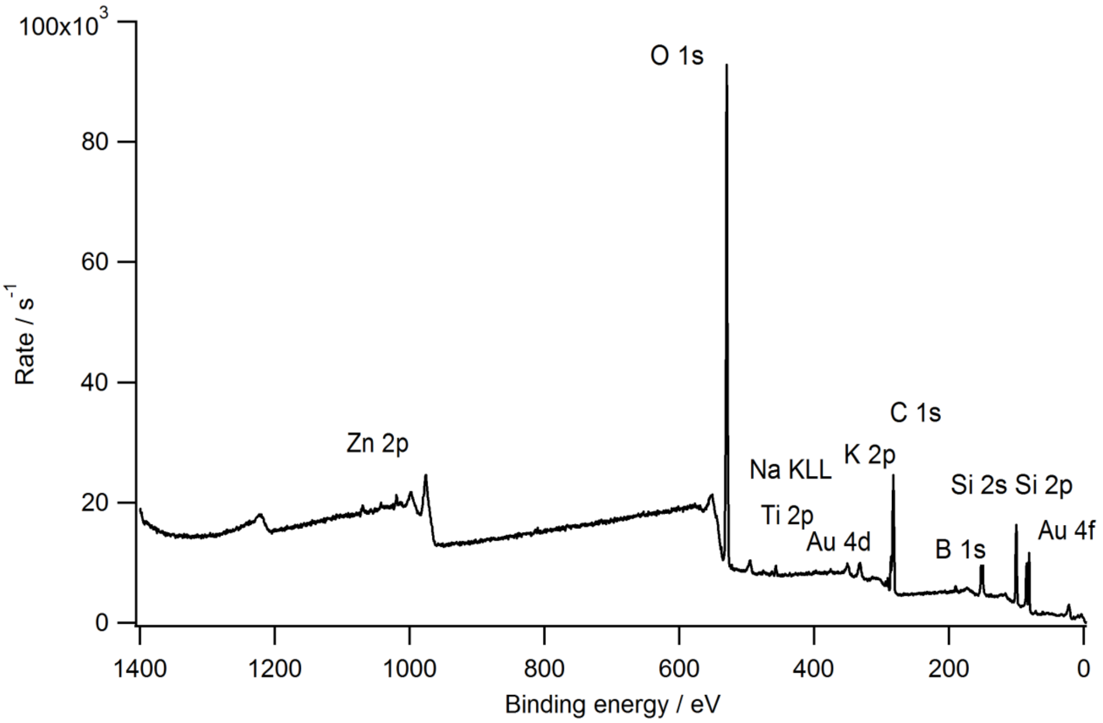

Figure S9: A low resolution wide scan XP spectrum over the full energy range, 1400 - -5 eV binding energy for the Au NP in polymer matrix. Labels indicate the principle photoelectron peaks detected. Since no Cl was observed in the wide scan spectrum, it is reasonable to assume that no gold chlorides from the starting material were present.

3D composite structures

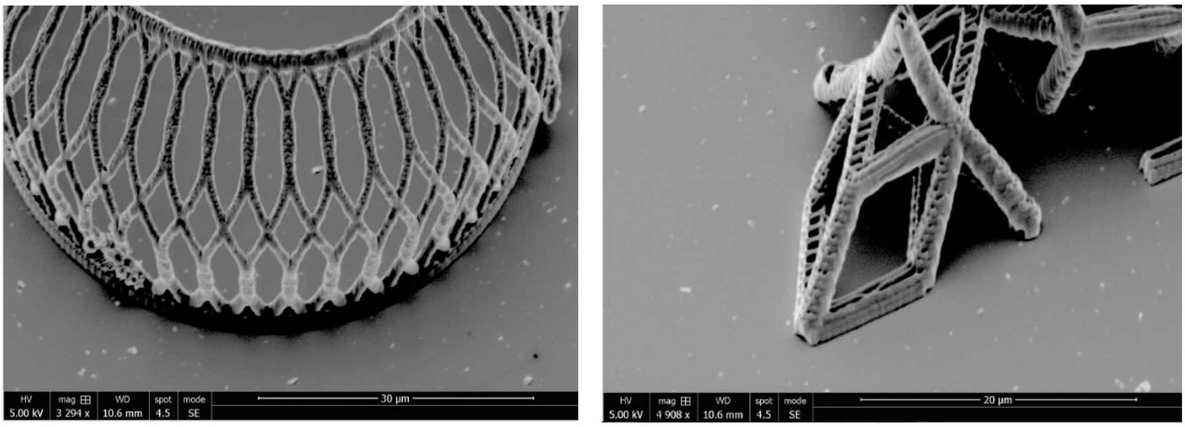

(a) (b)

Figure S10: Tilted SEM images showing the ring structure and pyramid structure shown in Figure 6(d) and (e). Please note the contrast was adjusted to show more detail of the surface morphology.

UV-vis spectroscopy:

Visible light of Thorlabs OSL 1-EC Fiber Illuminator was collimated and focus with an Olympus Plan 10x Objective. The focus spot size on the sample was ~200um. The sample was mounted on a XYZ translation stage with micrometre drives and placed at the focal point. Light after the sample was collected with another Olympus Plan 10x Objective and lens and then focused into an Andor Shamrock 303i spectrometer with a grating of 300l/mm and measured with an Andor Newton 940U CCD. Due to limited spectral coverage, one spectrum was obtained by combining two spectra centred at 560 nm and 700 nm respectively. Reference was measured at a position of substrate without polymer/gold structure and signal was measured at a position of substrate with polymer/gold structure. The spectrum is then calculated as,

$$Absorption = -\log {}^{I_{sig}}\!/_{I_{ref}}.$$

Four Au-containing samples and one none-Au sample were prepared for optical absorption analysis, each with a thin film of 1 mm x 1mm x 2 µm on glass substrate. The big thin film structure was made by stitching 100 (10x10 style) unit square structures, each with a size of 100 µm x 100 µm x 2 µm (due to the limited projection area of the Galvo scanner during laser fabrication). Therefore, the collected absorption signal came from 4-9 unit square structures, depending on the projection area of the light source on sample. For each sample we have examined different locations. As mentioned in the main text, local burning was sometimes observed, due to the plasmonic heating of the *in situ* generated Au nanoparticles. Although much effect has been tried to optimize the laser parameter setting to minimize this random effect, it is challenge to completely eliminate this phenomenon through 100 times unit structure repetition to reach mm areas. Therefore, minor shift in the location of the

absorption peak and strength was sometimes observed for different samples and different examining area.

References

1. Jiang, L. J.; Zhou, Y. S.; Xiong, W.; Gao, Y.; Huang, X.; Jiang, L.; Baldacchini, T.; Silvain, J. F.; Lu, Y. F., Two-photon polymerization: investigation of chemical and mechanical properties of resins using Raman microspectroscopy. *Optics Letters* **2014,** *39* (10), 3034-3037.

2. Burmeister, F.; Steenhusen, S.; Houbertz, R.; Zeitner, U. D.; Nolte, S.; Tunnermann, A., Materials and technologies for fabrication of three-dimensional microstructures with sub-100 nm feature sizes by two-photon polymerization. *J. Laser Appl.* **2012,** *24* (4).

3. Liu, Y.; Hu, Q.; Zhang, F.; Tuck, C.; Irvine, D.; Hague, R.; He, Y.; Simonelli, M.; Rance, G.; Smith, E.; Wildman, R., Additive Manufacture of Three Dimensional Nanocomposite Based Objects through Multiphoton Fabrication. *Polymers* **2016,** *8* (9), 325.
